# Composition of Bacterial Assemblages in Different Components of Reed Warbler Nests and a Possible Role of Egg Incubation in Pathogen Regulation

**DOI:** 10.1371/journal.pone.0114861

**Published:** 2014-12-10

**Authors:** Hanja B. Brandl, Wouter F. D. van Dongen, Alžbeta Darolová, Ján Krištofík, Juraj Majtan, Herbert Hoi

**Affiliations:** 1 Konrad Lorenz Institute of Ethology, Department of Integrative Biology and Evolution, University of Veterinary Medicine Vienna, Savoyenstrasse 1a, 1160 Vienna, Austria; 2 Institute of Zoology, Slovak Academy of Sciences, Dúbravská cesta 9, 845 06 Bratislava, Slovakia; University of Akron, United States of America

## Abstract

Bacteria play a central role in animal health. Yet, little is known about the acquisition of bacteria and the extent to which bacteria are acquired from different environmental sources. For example, bird nests host diverse bacteria associated with the eggs, nestlings and nesting material, but previous research has typically focussed on only a limited number of nest components at a time. It therefore remains unknown to what extent bacteria are transmitted between these components. Using both molecular and culture techniques, we characterised nest-associated bacterial assemblages throughout the entire nesting cycle of reed warblers by sampling bacteria on eggs before and during incubation, within nestling faeces, and on the nesting material of post-breeding nests. We found that bacterial assemblages clustered by nest component. Yet some overlap existed between nest components, suggesting that bacterial transmission across components is likely to occur. Eggs and nestlings from the same nest harboured more similar bacteria than expected by chance, suggesting an influence of environment or genetics on bacterial assemblages. Bacterial loads were not lower on incubated eggs. Instead, incubation was associated with a change in the structure of assemblages, including a decrease in potentially-harmful Gram-negative bacteria. In addition we show for the first time, that incubation is associated with the complete extinction of harmful haemolytic bacteria. Overall, our study appears to be the first to demonstrate differences in bacterial assemblages between bird nest components. In addition, we highlight the complexity of nest bacterial assemblages and provide new insights into the benefits of incubation.

## Introduction

Animals harbour diverse bacteria, which are often central to host health and fitness. The bacterial signatures of individual animals are composed predominantly of commensal or beneficial bacteria that are often involved in key processes such as digestion and immune function [1]–[3]. However, animals also host pathogenic bacteria that can be detrimental to host health under certain conditions [4]. In addition to the effects of individual bacterial species on hosts, the structure and diversity of entire bacterial assemblages are known to have broad implications on host health [5]. The profound effects of bacteria on animal health and fitness has led to increasing research focussing on the causes and implications of microbial diversity in animals, including host age [6], [7], nutrition [8]–[11], genotype [12], [13] and phylogeny [14]. Further, various modes of vertical and horizontal transmission are known to influence bacterial assemblages [15]–[20]. Animals exchange bacteria with their immediate surroundings, and the bacteria colonising animal dwellings, such as nests, burrows or hollows [21], [22] may therefore play a key role in transmission pathways and have important health consequences for hosts. Lastly, dwelling-associated bacteria are of economic importance as they pose a potential risk for disease transmission in livestock and poultry breeding [23]–[25].

The nests of wild birds harbour diverse bacteria, which may have profound effects on host fitness by influencing nesting success (e.g. egg and nestling viability) [22], [26], and the gastrointestinal microbiota of nestlings [27]. Various behaviours have therefore evolved to control pathogenic microbiota in the nests, including the lining of nests with leaves [28], [29] or feathers [30] that have antimicrobial properties. In addition, the incubation of eggs is known to be an important means to regulate the bacteria present on eggs. Incubation is thought to impair the growth of microbes on egg shells by reducing humidity and permitting the application of antimicrobial substances to the eggshells. This in turn may prevent trans-shell infection of pathogens which can dominate when eggs are exposed over longer periods and can ultimately harm the embryo [26], [31]–[33].

Despite these advances in our understanding of nest-associated bacteria, little is known regarding how bacterial assemblages are acquired, and how they differ and are transmitted between the various nest components, such as nesting material, eggs and nestlings. Past studies have suggested that the acquisition of bacteria within nests may have both a genetic and environmental component [6], [8], [34]. However, studies on the bacterial assemblages present on multiple nest components within the same nest are lacking. This information is crucial to gain insights into how bacteria are transmitted within bird nests and how this exchange of bacteria between nest components may influence bird health and fitness.

We sampled bacterial assemblages from different sources associated with the nests of reed warblers (*Acrocephalus scirpaceus*), including the surface of eggs prior to and during incubation, nestling faeces and nesting material (hereafter referred to as “nest components”). The contrasting environmental conditions associated with these nest components may favour the growth of different bacterial taxa. Sampling at various stages during the nesting cycle allowed us to assess the degree of overlap in bacterial assemblages between nest components and hence how much bacterial transmission may be occurring. We further documented the prevalence of various bacterial groups across nesting stages, allowing us to infer in which periods of the nesting cycle infections with potential pathogens could occur. For example, sampling the bacteria of nesting material immediately after fledging occurred allowed us to assess which potential pathogens older nestlings may have been exposed to. Lastly, we tested the general prediction that incubation influences the microbiota found on the surface of eggs. We adopted two complementary approaches to characterise bacterial communities. First we used a genetic technique, Automated Ribosomal Intergenic Spacer analysis (ARISA) [35], to characterise overall bacterial diversity and similarity between nest components. As ARISA does not provide information on the phylogenetic identity of bacteria, we additionally cultured bacteria to gain a more comprehensive overview on the characteristics of bacterial assemblages within reed warbler nests.

## Materials and Methods

### Study area and species

This study was conducted at Vel’ké Blahovo, Slovakia (48°03′09′′N, 17°35′38′′E) from April to July in 2012. The area contains three fishponds covering approximately 70 ha. The ponds are partly covered with marsh stands consisting of common reed (*Phragmites australis*), lesser reedmace (*Typha angustifolia*), great reedmace (*T. latifolia*) and *Carex* spp. Temperatures during the study period ranged from a mean monthly minimum temperature of 11.8°C to a mean monthly maximum temperature of 25.0°C. The total amount of precipitation per month ranged from 24.0 mm to 95.6 mm [36].

The seasonally monogamous reed warbler is a long-distance migrant that overwinters in central Africa. At the beginning of April, reed warblers start to arrive at the study area for breeding. Nests are typically constructed from reed flowers from the previous year. Blades of grass, seed hair from various trees and spider webs are then added to the deep, open, cup-shaped nests [37]. Females lay eggs in the early morning hours, in one-day intervals. Clutch incubation usually starts after the laying of the third egg and continues for a period of 11–14 days. Nestlings usually fledge when 10–13 days old [38]. During the study period, the average clutch size per nest was 4.0±0.1 SE eggs (means ± standard errors throughout; range: 3–5) and the average number of fledglings was 3.3±0.2 fledglings (range: 1 to 5).

### Field work

The commencement of the breeding season was determined by the detection of singing males. We then searched for nests under construction. Nests are typically constructed over 5–7 days. Once finished, the nests were inspected almost daily in order to determine the onset of egg-laying. Bacterial samples were collected at different stages of the nesting cycle, over a period of 10 weeks. First, sampling of bacteria from preincubation eggs occurred in the morning of the day that the second egg was laid. This ensured that incubation had not yet commenced. To sample eggs at this stage, we removed both eggs from the nest with sterile gloves and swabbed the surface of each egg with a sterile cotton swab, soaked in sterile water, for 17 seconds (i.e. the average time required to swab the entire surface of one egg). One swab per egg was used. The swabs were then stored in transport medium (Transport viscose swab with Amies transport medium, Sarstedt). Next, we sampled eggs during the incubation period on day 10 after the laying of the first egg. The same sampling method was repeated, but this time the surfaces of all eggs within the nest were sampled with a single swab for 17 seconds.

Faecal samples of two nestlings were collected when nestlings were between five and seven days old. For sampling, the nestlings were placed on a sterile plastic sheet and after defecation the faeces was collected with sterile tweezers and stored in an Eppendorf tube filled with 1 ml of Phosphate-buffered saline (PBS). On the day that the last nestling fledged, or at latest the day after, we placed the deserted nests in sterile plastic bags. In the laboratory the inner wall of each nest cup was moistened with 3 ml of sterile water and swabbed for 10 seconds. The nesting material was sampled after nestlings had fledged to avoid disturbance at a sensitive stage and the possibility of nest abandonment. However, nestlings occasionally defecate in the nests, despite their parents’ efforts of keeping the nest clean. This therefore allowed us to explore to what extent bacteria present in post-breeding nests are associated with faecal bacteria of the nestlings. In addition, it allowed us to document which potential pathogens nestlings are likely to be exposed to prior to fledging. Finally, control samples were intermittently taken throughout the sampling period to ensure that our samples were not contaminated with non-nest bacteria. Controls were taken by exposing moistened swabs to the air and storing them in transport medium as described above. The PBS and water which we used for sampling were also used as controls, to ensure that these solutions were not contaminated.

### Laboratory work

In the laboratory, we transferred each swab from the transport medium into a tube containing 2.5 ml of PBS. The swab was thoroughly mixed within the tube to ensure that the bacteria became suspended within the solution. The faecal samples were diluted with PBS according to their weight, at a dilution of either 1∶25 or 1∶10, and then vortex mixed. We then used some of each sample for bacterial culturing and the remaining sample for DNA extraction.

#### Genetic analyses

Bacterial DNA was extracted from the samples using a Qiagen DNeasy Blood & Tissue Kit, following the manufacture’s protocol for the purification of total DNA from bacteria. This included a pretreatment for Gram-positive bacteria, as these bacteria have thicker cell walls than Gram-negative bacteria. To characterise the assemblages of both Gram-positive and Gram-negative bacteria present in each sample we used ARISA [35]. This molecular technique exploits the extreme interspecies variability in the length of the intergenic spacer (IGS) lying between the conserved 23S and 16S genes in the bacterial ribosomal operon. ARISA involves the PCR amplification of the bacterial IGS using a fluorescently-labelled primer and subsequent high-resolution electrophoresis in an automated system. Assemblages are therefore characterised by a series of electrophoretic peaks that vary according to the length of the amplified IGS fragment of each bacterial OTU (operational taxonomic unit).

An advantage of ARISA is that it can be used to rapidly quantify bacterial diversity within samples, as well as similarities between assemblages. However, for a number of reasons, ARISA can only estimate bacterial diversity and not reveal complete assemblage structure. First, unrelated OTUs may share the same IGS fragment length (i.e. one electrophoretic peak may represent multiple bacterial OTUs). In addition, an estimated 8% of bacteria will not be detected by ARISA given that they either do not have their 23S and 16S rRNA genes organised in an operon or have IGS lengths that are too large to be detected by the automated sequencer [39]. Last, some bacterial species may have multiple operons (i.e. several electrophoretic peaks may represent a single species [36]). Despite these caveats, ARISA remains a relevant and important tool to characterise bacterial assemblages [18], [40]–[43], especially when coupled with other microbiological techniques, such as bacterial culturing.

We performed ARISA following the protocols outlined in [7]. Each ARISA profile was subsequently viewed in Genemapper 4.0 (Applied Biosystems) and the fragment length and intensity of each electrophoretic peak was recorded. Peak intensity served as a proxy for the abundance of each OTU within each assemblage [44], which was subsequently used to estimate bacterial diversity (see below). The DNA fragment amplified by our primers consisted of the bacterial IGS, as well as approximately 20 bp and 130 bp of the 16S and 23S rRNA genes, respectively. As bacteria with IGS lengths of less than 150 bp are unknown [7], [35], [45], we only considered peaks of fragments longer than 300 bp. Electrophoretic peaks of less than 300 bp were considered PCR artefacts.

#### Cultivation of bacteria

Bacterial suspensions were serially diluted in order to find the suitable concentration for culturing. The dilutions ranged from undiluted samples to dilutions of 10^12^, as samples from different sources showed a high variation in bacterial concentrations. The dilution that yielded between 30 and 200 colony forming units (CFU) per plate was used for the final CFU count. 100 µl of each dilution was spread-plated on two plates with different media: (1) non-selective medium (Columbia agar with sheep blood 7%, Oxoid), which allowed the distinction between haemolytic and non-haemolytic bacteria, and (2) selective medium (Brilliance UTI, Oxoid), which allowed the detection of specific bacterial taxa (i.e. *Escherichia coli*, *Enterococcus* spp., coliforms, *Proteus* spp., *Morganella* spp., *Providencia spp.*, *Pseudomonas* spp., *Staphylococcus* spp., *Streptococcus* spp., *Staphylococcus saprophyticus*). These taxa are known urinary tract pathogens in humans, but are also associated with a variety of infections in birds [46]–[53]. As the sampling process was standardised, CFU growth on both media indicated total bacterial counts, after arithmetically controlling for the use of different dilutions. Plates were incubated for 24 h at 37°C. Afterwards, we screened and quantified the CFUs for haemolytic activity on the non-selective sheep blood agar plates. On the selective plates, we taxonomically classified the CFUs according to their colour, following the manufacturer’s protocol. Three groups could be clearly identified: (1) *Enterococcus* spp., (2) coliforms, and (3) *Staphylococcus* spp. and *Streptococcus* spp. Another group was formed by merging two taxa, as they could not be clearly distinguished by colour: (4) *E. coli* and *Staphylococcus saprolyticus.* The other bacterial taxa (*Proteus* spp., *Morganella* spp., *Providencia* spp., and *Pseudomonas* spp.) could not be identified with certainty and were therefore not included in the analysis.

### Statistical analyses

#### ARISA data

The structure and characteristics of bacterial assemblages were analysed using the community ecology software PRIMER v6.1.6 [54]. Assemblage diversity was calculated for each bacterial sample using the total number of species detected (S), the Shannon diversity index (H′ = −Σρ*_i_*lnρ*_i_*, where ρ represents the proportion of the total abundance arising from the *i*th OTU) and the Simpson diversity index (1-λ′ = 1–[(ΣN*_i_*(N*_i_*-1)/N(N-1)], where N*i* is the abundance of OTU *i*). Before the diversity estimates were calculated we controlled for intersample differences in DNA extraction efficiency and PCR amplification success by dividing the intensity of each peak within an ARISA profile by the sum of all peak intensities within that profile [19].

We calculated a zero-adjusted resemblance matrix for all samples using the Bray-Curtis similarity measure. This matrix was then used to estimate the mean similarities of samples within and between the four nest components. Similarities within and between nest components were visualised using a non-metric multi-dimensional scaling analysis (nMDS). The fit of the data was assessed via the stress values associated with the nMDS, with a stress of less than 0.2 being acceptable. Data were mapped into both two- and three-dimensional space. The variability in bacterial assemblage structure of each nest component was assessed using the multivariate dispersion index.

As we sampled two preincubation eggs per nest (N = 8 nests) and faecal samples from two nestlings per nest (N = 9 nests) we were able to test whether eggs/nestlings within nests had more similar assemblages compared to eggs/nestlings between nests (i.e. whether eggs or nestlings within the same brood were more likely to share bacteria). This was done using the software programme PERM [55]. PERM allows comparison of the mean similarity of a dyad originating from one nest with that expected by random allocation of the samples to different nests. PERM uses matrices of a pairwise relatedness statistic (“Sxy” – which corresponds to Bray-Curtis similarity in this study) and calculates the sum of all Sxy values (i.e. Bray-Curtis sums) for each dyad. This value is then compared to a distribution of Sxy sums generated from randomly assigning samples to nests. 1,000 randomisations were used.

#### Cultured bacteria

After identifying and counting the CFUs on the plates we calculated the number of CFUs per mL of each sample. We log-transformed all abundance data due to the high variation in the number of bacteria in our samples (range – blood agar plates: 10–1.2×10^9^ CFUs, UTI plates: 10–7.5×10^8^ CFUs). All analyses comparing means across nest components (e.g. cultured bacterial loads, ARISA OTU diversity) were conducted using generalised linear mixed models to account for the non-independence of samples collected from the same nest. Distributions followed either a normal or gamma distribution (with an identity or logarithm link, respectively). We found that the abundances of cultured bacteria varied with sampling date within some nest components (S1 Table). We therefore included sampling date as a covariable in all GLMMs that focussed on cultured bacteria. In contrast, we found no association between sampling date and any measure of ARISA OTU diversity for any nest component (0.207<p<0.950). We therefore did not include sampling date in the GLMMs focussing on the ARISA data. All analyses of the cultured bacteria data were conducted in IBM SPSS Statistics 20, as were additional statistical analyses of the ARISA data.

### Ethics Statement

All field procedures were conducted according to the respective legislation of the Slovak Republic and following the conditions and guidelines approved by the Ministry of Environment of the Slovak Republic (permit number 1443/04).

## Results

### ARISA analyses

#### Prevalence of operational taxonomic units

We detected a total of 196 OTUs ranging in size from 300 to 1182 bp. Seventeen OTUs were present in all four nest components, 45 were present in three of the nest components, whilst 64 OTUs were present in only a single component of the nest. OTU prevalence was highly variable within nest components, with some OTUs being very common and the majority being less prevalent (S1 Figure). For example, only 10 of the 166 OTUs found in the nesting material were detected in over 50% of samples, while 55 OTUs had a prevalence of less than 10%. The different nest components significantly differed in number and diversity of OTUs per sample (Table 1). Nesting material harboured a greater number and diversity of OTUs than both eggs and faecal samples (Table 1).

**Table 1:** Differences in bacterial diversity and abundance between nest components.

	Preincubation	Incubated	Nestling faeces	Nest material	F	P value
**ARISA**						
**N**	31	13	25	27		
**Number of OTUs**	8.5±0.6	8.2±1.0	9.6±0.9	31.7±2.7	40.285	<0.001
**Shannon diversity**	1.6±0.1	1.8±0.2	1.4±0.1	2.6±0.1	21.006	<0.001
**Simpson diversity**	0.73±0.03	0.78±0.04	0.63±0.03	0.86±0.03	10.605	<0.001
**Cultured bacteria**						
**N**	55	25	50	24		
**Blood Agar abundance**	3.9±0.2	3.0±0.2	5.7±0.2	7.6±0.3	58.717	<0.001
**Percentage Haemolytic**	3.3±12.4	0.0±0.0	7.1±17.7	1.8±5.0	1.587	0.209
**UTI abundance**	3.2±0.2	3.0±0.2	5.5±0.2	7.5±0.2	99.627	<0.001

Differences between the four nest components in 1) diversity of OTUs per sample using ARISA and 2) abundance of bacteria per sample quantified using culturing techniques. Data are presented as predicted means ± standard error. Cultured bacteria abundance means were calculated using log_10_ transformed data. Proportions of haemolytic bacteria are stated as percentage. “Preincubation” and “Incubated” indicate eggs sampled before and during the incubation period, respectively. Generalised linear mixed models were used due to the non-independence of samples collected from the same nest.

#### Differences in assemblages between nests and nest components

**Figure 1:**
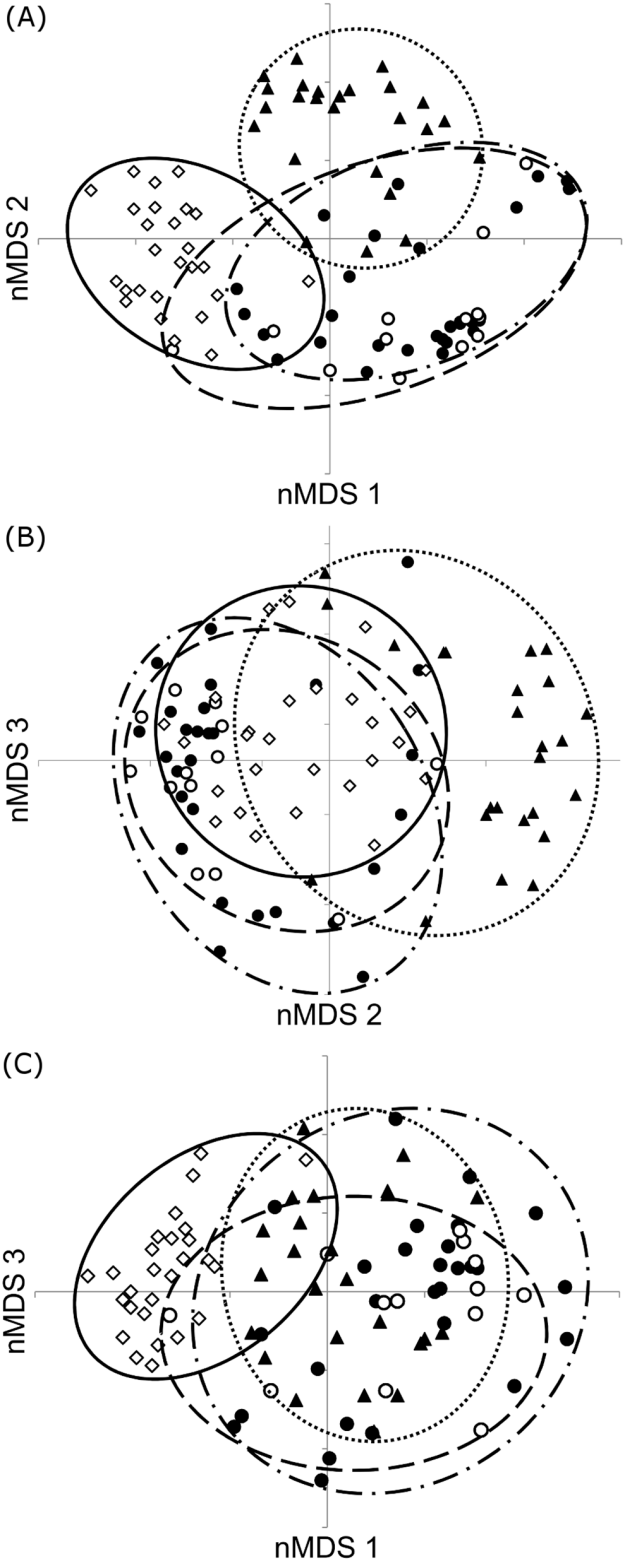
Differences in bacterial assemblages between the nest components. Non-metric multi-dimensional scaling along A) the first and second axis, B) the second and third axis and C) the first and third axis, displaying differences in bacterial assemblages between the four nest components including preincubation eggs (closed circles and dash dot line), incubated eggs (open circles and long dashed line), nestling faeces (closed triangles and dotted line) and nesting material (open diamonds and a solid line).

Bacterial assemblages sampled from preincubation eggs of the same nest tended to be more similar to each other than expected by chance (observed similarity sum: 249.4; expected similarity sum: 198.8±13.2; N = 8; p = 0.069). Similarly, within-nest similarity of bacterial assemblages sampled from nestling faeces was greater than inter-nest assemblage similarity (observed similarity sum: 217.8; expected similarity sum: 168.7±7.3; N = 9; p = 0.011). The nMDS revealed pronounced differences in the structure of bacterial assemblages between the four different nest components. The stress of the two-dimensional analysis was 0.26, suggesting that the variation in the data could not be reliably captured in two dimensions. The stress of the three-dimensional analysis was acceptable (stress = 0.19). We found strong clustering of assemblages by nest component. Despite the close proximity of nesting material with the nestlings, we found rather little overlap in their bacterial assemblages (Fig. 1, Table 2). In contrast, we detected a strong overlap in the assemblages on eggs before and during incubation (S2 Figure). The multivariate dispersion indices indicated that intersample variation in assemblage structure was lowest in the nesting material samples (multivariate dispersion index = 0.77). Assemblages on eggs sampled before and during incubation displayed much higher variability (multivariate dispersion indices = 1.13 and 0.96 respectively), similar to the variability found in the assemblages in nestling faeces (multivariate dispersion indices = 1.1).

**Table 2:** Mean Bray-Curtis similarities between nest components.

	Preincubation	Incubated	Nestling faeces	Nesting material
**N**	22	13	16	27
**Preincubation**	27.5±4.1			
**Incubated**	28.4±4.1	30.5±5.1		
**Nestling faeces**	14.5±1.3	13.6±1.6	24.0±2.9	
**Nesting material**	12.7±1.4	15.1±2.4	13.8±1.6	31.4±1.7

Data are presented as means ± standard error. “Preincubation” and “Incubated” indicate eggs sampled before and during the incubation period, respectively.

### Cultured bacteria analyses

#### Differences in assemblages between nest components

**Figure 2:**
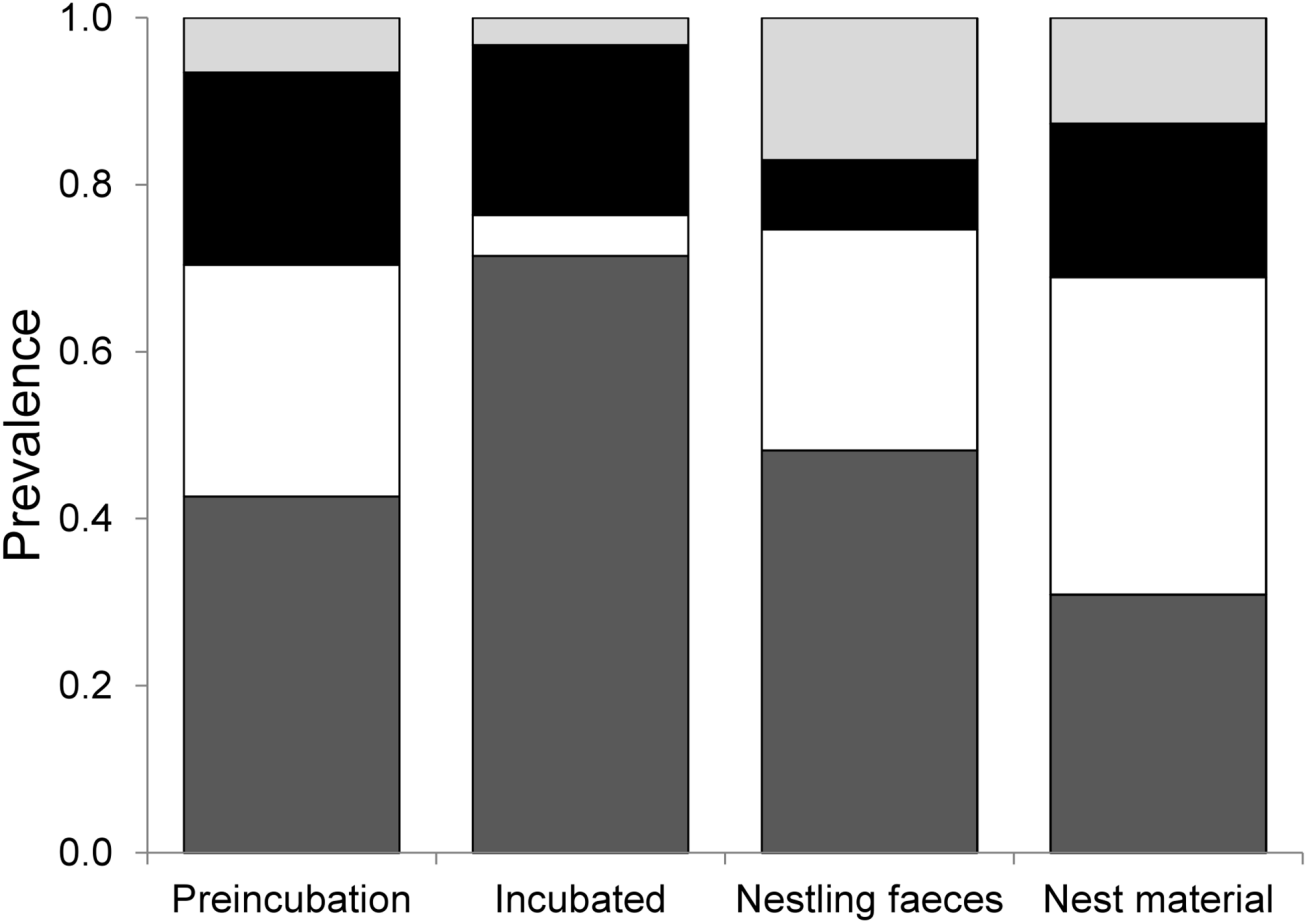
Prevalence of cultured bacteria in different nest components. Prevalence of cultured bacteria between nest components, including *Enterococcus* spp. (dark grey), coliforms (white), *Staphylococcus/Streptococcus* spp. (black) and *Escherichia coli/Staphylococcus saprophyticus* (light grey).

The composition of bacteria between each nest component varied markedly. *Enterococcus* and coliforms spp. were the most abundant bacteria detected, comprising up to 76.4% of the bacteria cultured from each nest component. Nesting material was dominated by coliform bacteria, while the remaining three nest categories were dominated by *Enterococcus*. *E. coli/Staphylococcus saprophyticus* was most commonly detected in nestling faeces and nesting material and was rare on the egg shells (Fig. 2). The dominance of *Streptococcus* and *Staphylococcus* tended to be similar among the nest components, except for a lower abundance in nestling faeces. Nesting material contained the highest abundance of bacteria, for both the blood agar culture and the UTI culture, while the lowest abundance of bacteria was found on the eggs (Table 1).

**Figure 3:**
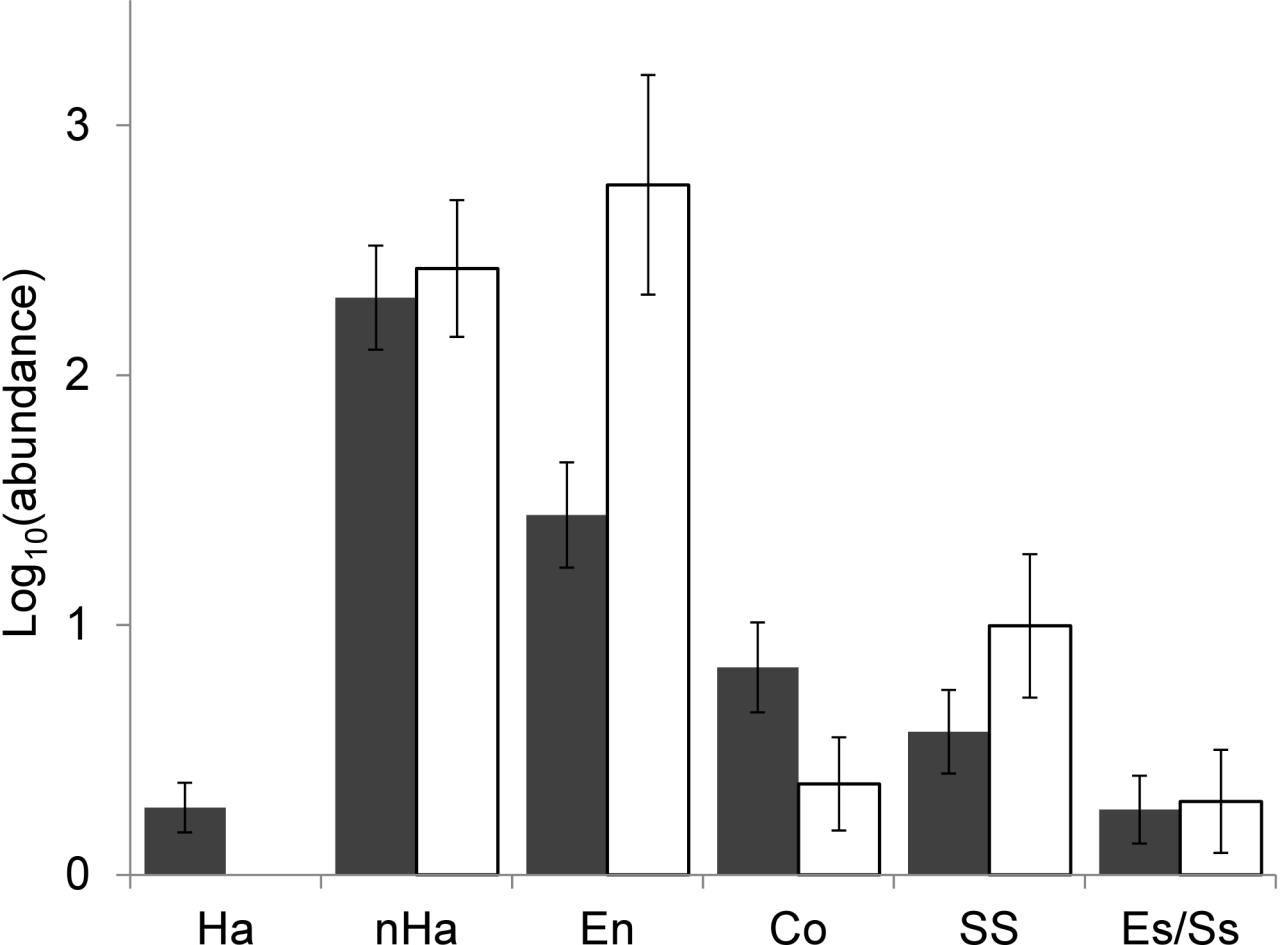
Abundance of cultured bacteria in preincubation and incubated eggs. Abundance of cultured bacteria sampled from eggs before and during incubation, including haemolytic bacteria (Ha), non-haemolytic bacteria (nHa), *Enterococcus* (En), Coliforms (Co), *Streptococcus*/*Staphylococcus* (SS) and *Escherichia coli/Staphylococcus saprophyticus* (Es/Ss). Bars represent predicted means ± SE from GLMMs, including sampling date as a covariate.

Overall, incubated eggs did not differ from preincubation eggs in terms of UTI bacterial loads (GLMM: nest component - F_1,72_ = 1.706, p = 0.196, date - F_1,72_ = 41.155, p<0.001). However, the structure of the assemblages did change during incubation (Fig. 2), including a loss of all haemolytic bacteria (Table 1). Haemolytic bacteria comprised a mean of 3.7±12.8% of the bacteria found in each nest component. Although 3.3±12.4% of bacteria on the preincubation egg shells were haemolytic, we detected no haemolytic bacteria on incubated eggs (Table 1). Haemolytic bacteria were most dominant in nestling faeces (7.1±17.7%), while 1.8±5.0% of bacteria in the nesting material were haemolytic (Table 1). Loads of non-haemolytic bacteria (GLMM: nest component - F_1,97_ = 0.195, p = 0.660, date - F_1,97_ = 65.261, p<0.001), *E. coli*/*Staphylococcus saprophyticus* (GLMM: nest component - F_1,72_ = 0.018, p = 0.895, date - F_1,72_ = 0.244, p = 0.623) and *Streptococcus*/*Staphylococcus* (GLMM: nest component - F_1,71_ = 2.279, p = 0.136, date - F_1,71_ = 7.484, p<0.001) did not differ between incubated and unincubated eggs (Fig. 3). In contrast, incubated eggs harboured more *Enterococcus* (GLMM: nest component - F_1,72_ = 11.757, p = 0.001, date - F_1,72_ = 5.925, p = 0.017) and tended to harbour fewer coliforms (GLMM: nest component - F_1,72_ = 3.551, p = 0.064, date - F_1,72_ = 10.364, p = 0.002; Fig. 3).

## Discussion

We have shown here that variation in the bacterial assemblages found within reed warbler nests is complex, with unique microbial signatures being detected for each nest component. The high number of bacterial OTUs occurring in low prevalence may be a crucial factor in forming the distinct bacterial signature of individual components. Almost one-third of bacterial OTUs were detected in either three, or all four, of the nest components, suggesting that bacterial transmission between components is likely to occur. In addition, components from the same nest shared more bacteria than components from foreign nests. Finally, egg incubation was not associated with an expected decrease in bacterial loads. Instead, the structure of the assemblages changed during the incubation period, with a reduction in potentially harmful bacterial groups, such as haemolytic bacteria. Overall, our results provide unique insights into the development of the bacterial signatures of each stage of the nesting cycle and highlight the benefits of egg incubation for coping with bacterial pathogens.

The similarity in bacterial assemblages on eggs and nestlings originating from the same nest adds to a growing body of evidence that individual nests harbour unique bacterial assemblages [6], [8], [34]. While our study design does not allow us to distinguish between genetic and environmental effects, a partial cross-fostering experiment in two tit species (*Parus major* and *Cyanistes caeruleus*), demonstrated that nestlings shared more gastrointestinal bacteria with foster- than genetic siblings [56]. This result suggests that rearing environment may have a stronger influence on the bacteria hosted by nestlings rather than host genotype.

The bacterial assemblages within each nest component were relatively divergent, yet we still detected between 12.7% and 28.4% overlap between assemblages. Although our broad community-scale approach cannot detect the transmission of OTUs, the amount of overlap between the assemblages of each component suggests that bacterial transmission between components is likely to occur to some extent.

Interestingly, we only found 13.8% similarity in the bacteria assemblages present in nestling faeces and on the nesting material. This is despite the nests being sampled immediately after fledging occurred, when the nest had presumably been in contact with nestling faeces over an extended period. As we sampled nestlings between days 5–7 after egg hatching, but nesting material after the nestlings had fledged, it is possible that this relatively low overlap may, in part, be explained by changes in the gastrointestinal bacteria of the nestlings as they aged [7], and in the bacteria on nesting material after the sampling of the nestling faeces. We may therefore have underestimated the true amount of overlap between the two nest components. Despite this *E. coli/Staphylococcus saprophyticus* were much more common in nestling faeces and nesting material than on the other components, suggesting that these species may have been transmitted from nestling faeces to the nesting material. Our results also suggest that the majority of bacteria found within nesting material was acquired from other environmental sources, such as from the original source of the nesting material itself or from the warbler parents, whose saliva [20] and plumage [57]–[59] presumably carry a high diversity of bacteria. The fact that the nesting material showed the highest abundance and diversity of bacteria supports the idea that it harbours bacteria from multiple sources.

Diverse factors may have reduced the similarity in bacterial assemblages between nest components, despite the close contact of the components over an extended period. First, nest sanitation may reduce the amount of transmission of faecal bacteria to the nesting material. For example, parents remove nestling faecal sacs from the nest in this species (Darolová, personal observation). In addition, the mucous covering of the sac itself likely serves as a bacterial barrier preventing contact between faecal bacteria and the nesting material [60]. However, not all attempts to remove faecal sacs from the nests were successful when, for example, older nestlings attempted to remove faecal sacs from the nests themselves or when parents removed multiple faecal sacs at the same time (Darolová, personal observation). Therefore a certain amount of bacterial transmission from nestling faeces to the nest was likely to occur.

A second factor contributing to the low overlap between components is that bacteria are sensitive to many external factors such as pH, oxygen availability and salinity [61] and a bacterial species from one source may not succeed in colonising a different environment. Third, interspecific mutualistic and competitive interactions between bacteria are likely to occur. Therefore, an environment that is already colonised by a certain bacterial community may make it more challenging for other taxa to colonise the same substrate [62]–[64]. Last, eggs have antibiotic properties that reduce the likelihood of colonisation by potentially pathogenic environmental bacteria [65]–[67].

One well-studied mechanism for reducing pathogenic infection of eggs is incubation.

Incubation is known to be effective in decreasing humidity [68] and thereby reducing the microbial diversity on eggs [33], [69]–[71] and the potential for trans-shell infection of pathogens [31], [70]. In our study, incubated eggs did not have lower bacterial loads than preincubation eggs, but instead the composition of the assemblages changed. This pattern has also been observed in other bird species [71]–[73]. While our community analyses based on the genetic data clearly demonstrate a high overlap in bacterial assemblages of eggs prior to and during incubation, our culturing results revealed that a shift in the proportions of specific bacterial taxa occurred on the egg shells during the incubation period. For example, we detected a significant decrease in the abundance of coliforms, a group of Gram-negative bacteria. Although Gram-positive bacteria often dominate the surface of eggshells, Gram-negative bacteria may be better equipped to survive the internal conditions of the egg and are commonly found in rotten eggs [74]. The decrease in coliforms that appears to be associated with incubation in our study could therefore be crucial in the preservation of embryo health.

In contrast, the abundance of Gram-positive cocci, (predominantly *Enterococcus spp*., but also *Streptococcus spp.*/*Staphylococcus spp.)* increased during incubation, even though *Enterococcus spp*. were already the most abundant microbial taxon on the eggshell before incubation. *Enterococcus* is a genus of Gram-positive lactic acid bacteria that are usually non-haemolytic and are commonly found in the gut microbiota. Many species of *Enterococcus* are known to be beneficial. For example, *E. faecium* promotes growth in pied flycatcher (*Ficedula hypoleuca*) nestlings, probably due to its competitive interactions with pathogenic species, such as *E. faecalis* [75]. In addition, *Enterococcus* species are commonly used as probiotics to increase animal productivity [76].

An additional striking observation that was associated with incubation was the complete extinction of haemolytic bacteria. We detected haemolytic bacteria on the nesting material, in the nestling faeces and on the eggs prior to, but not after, incubation. In contrast, the abundance of non-haemolytic bacteria did not decrease during incubation. Haemolytic bacteria are considered pathogenic due to their ability to damage red blood cells, which presumably causes harm to their host. For example, haemolytic activity was detected in almost a third of microorganisms that were isolated from bird eggs that had failed to hatch [77]. Haemolytic activity occurs in many bacterial taxa, and various haemolytic species, especially species of *Streptococcus*, are associated with diseases in birds [46], [78]–[81]. Our study is the first to suggest that the incubation of eggs is an important mechanism to reduce, or even completely eliminate, bacteria with harmful haemolytic properties.

Once hatched, the nestlings are likely to be exposed to pathogens within the nesting material. The presence of potential pathogens in nests has been previously documented [22] and bacteria in nests may affect nestling condition [82]. Our culturing revealed that nesting material harboured a higher proportion of coliforms and a lower proportion of *Enterococcus* spp. than both eggs and nestling faeces. Coliforms, a group of Gram-negative fermenters, can act as pathogens in birds and cause infectious diseases, such as enteritis, salpingitis, peritonitis [53] or coliform-septicemia [52], which is common in farmed poultry. Nestlings were therefore likely exposed to these potential pathogens immediately prior to fledging. Whether these bacteria affect the condition of warbler nestlings warrants further investigation.

Overall, our results highlight the diversity of bacteria found in bird nests and the potential complexity of bacterial transmission between nest components. Our data suggest that the various nest components, which represent different stages of nestling development, provide contrasting ecological conditions for bacterial growth, thus favouring the proliferation of different bacterial taxa. Nestlings therefore appear to be confronted with different challenges at the various stages of their development (i.e. within the egg during embryonic development and within the nest upon hatching). In addition to inter-component divergence in assemblages, intra-component variation was also high, with only approximately 30% similarity in assemblages within each component from different nests. This suggests nest-specific characteristics in determining the structure of bacterial assemblages and a strong influence of either the rearing environment or host genotype. Despite this high microbial variation, we also detected some overlap between the bacterial communities on nests, eggs and faeces, suggesting that bacterial transmission may be occurring between nest components, including pathogen transmission. Finally, we were also able to support the idea that incubation reduces the prevalence of certain pathogens. Most impressively, incubation was associated with the complete extinction of haemolytic bacteria, which pose a potentially severe threat to the health of the developing embryo.

## Supporting Information

S1 Figure **Prevalence of OTUs for different nest components.**
(DOCX)

S2 Figure **Differences in bacterial assemblages between the preincubated and incubated eggs.**
(DOCX)

S1 Table **Associations between cultured bacterial loads and sampling dates for each nest component.**
(DOCX)
