# Supplementary material for: Composition of Bacterial Assemblages in Different Components of Reed Warbler Nests and a Possible Role of Egg Incubation in Pathogen Regulation
Source: PLoS One. 2014 Dec 10;9(12):e114861. doi: 10.1371/journal.pone.0114861 (PMC4262450; doi:10.1371/journal.pone.0114861)
Supplement: S1 Figure — Prevalence of OTUs for different nest components. (DOCX) [file pone.0114861.s001.docx]

**Figure S1.** **Prevalence of OTUs** **for different nest components.** Prevalence of OTUs (i.e. percent of samples for which each OTU was detected) for a) preincubation eggs b) incubated eggs, c) nestling faeces and d) nest material. Each vertical bar represents an OTU and the position of each OTU is identical within each graph.

**Figure 1a**

**Figure 1b**

**Figure 1c**

**Figure 1d**
