# Supplementary material for: Composition of Bacterial Assemblages in Different Components of Reed Warbler Nests and a Possible Role of Egg Incubation in Pathogen Regulation
Source: PLoS One. 2014 Dec 10;9(12):e114861. doi: 10.1371/journal.pone.0114861 (PMC4262450; doi:10.1371/journal.pone.0114861)
Supplement: S2 Figure — Differences in bacterial assemblages between the preincubated and incubated eggs. (DOCX) [file pone.0114861.s002.docx]

**Figure S2.** **Differences in bacterial assemblages between the preincubated and incubated eggs.** Non-metric multi-dimensional scaling (stress = 0.16) along the first and second axis of three axes, displaying differences in bacterial assemblages between preincubated eggs (closed circles and dash dot line) and incubated eggs (open circles and long dashed line).

**
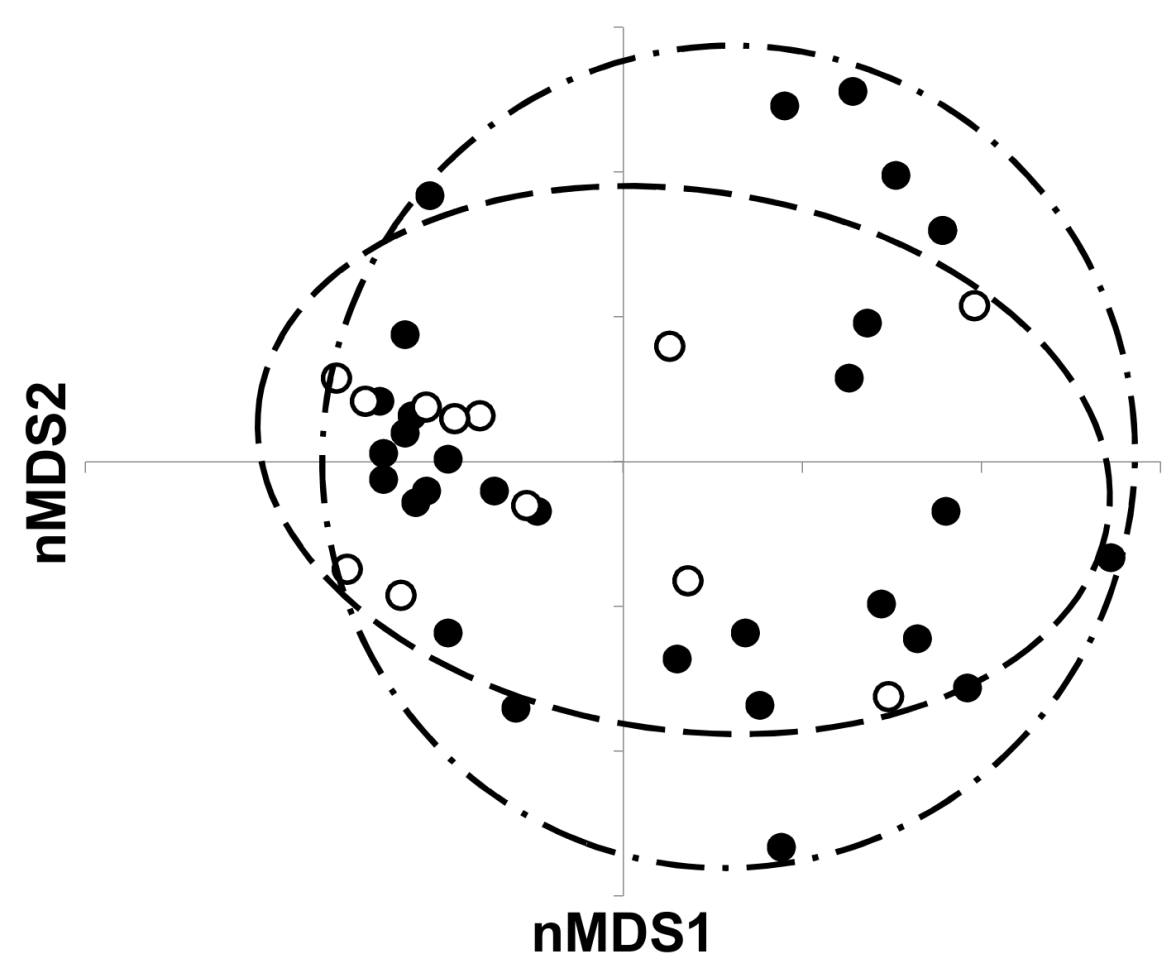
**
