# Supplementary material for: Composition of Bacterial Assemblages in Different Components of Reed Warbler Nests and a Possible Role of Egg Incubation in Pathogen Regulation
Source: PLoS One. 2014 Dec 10;9(12):e114861. doi: 10.1371/journal.pone.0114861 (PMC4262450; doi:10.1371/journal.pone.0114861)
Supplement: S1 Table — Associations between cultured bacterial loads and sampling dates for each nest component. (DOCX) [file pone.0114861.s003.docx]

**Table S1.** **Associations between cultured bacterial loads and sampling dates for each nest component**. The associations were calculated using generalised linear mixed models. The probability of finding x of y significant tests by chance alone was calculated via a Bernoulli process [[1](#_ENREF_1)].

| **Nest group** | **Bacteria** | **Coefficient** | **F^a^** | **p** |
| --- | --- | --- | --- | --- |
| Preincubation | Haemolytic | -0.016 | 3.619 (1,67) | 0.069 |
| Preincubation | Non-haemolytic | -0.047 | 72.521 (1,67) | **< 0.001** |
| Preincubation | *Enterococcus* | -0.014 | 1.626 (1,49) | 0.208 |
| Preincubation | Coliforms | -0.034 | 10.717 (1,49) | **0.002** |
| Preincubation | *Staphylococcus*/*Streptococcus* | -0.025 | 5.015 (1,48) | **0.030** |
| Preincubation | *E. coli/S. saprophyticus* | -0.002 | 0.023 (1,49) | 0.880 |
| Probability of obtaining 3 of 6 significant tests with p < 0.03 by chance alone: p = 0.0005 | | | | |
| Incubation | Haemolytic |  |  | Absent |
| Incubation | Non-haemolytic | -0.039 | 20.473 (1,29) | **< 0.001** |
| Incubation | *Enterococcus* | -0.030 | 10.783 (1,22) | **0.003** |
| Incubation | Coliforms | -0.007 | 0.527 (1,22) | 0.475 |
| Incubation | *Staphylococcus*/*Streptococcus* | -0.024 | 3.130 (1,22) | 0.091 |
| Incubation | *E. coli/S. saprophyticus* | -0.006 | 0.492 (1,22) | 0.491 |
| Probability of obtaining 2 of 5 significant tests with p < 0.003 by chance alone: p < 0.0001 | | | | |
| Nestling faeces | Haemolytic | 0.012 | 1.684 (1,48) | 0.201 |
| Nestling faeces | Non-haemolytic | 0.008 | 14.138 (1,47) | **< 0.001** |
| Nestling faeces | *Enterococcus* | 0.009 | 9.711 (1,48) | **0.003** |
| Nestling faeces | Coliforms | 0.007 | 0.650 (1,48) | 0.424 |
| Nestling faeces | *Staphylococcus*/*Streptococcus* | 0.023 | 4.703 (1,48) | **0.035** |
| Nestling faeces | *E. coli/S. saprophyticus* | 0.036 | 12.714 (1,48) | **0.001** |
| Probability of obtaining 5 of 6 significant tests with p < 0.035 by chance alone: p < 0.0001 | | | | |
| Nest material | Haemolytic | -0.007 | 0.192 (1,23) | 0.666 |
| Nest material | Non-haemolytic | 0.004 | 2.275 (1,22) | 0.146 |
| Nest material | *Enterococcus* | 0.004 | 0.980 (1,23) | 0.332 |
| Nest material | Coliforms | 0.003 | 0.058 (1,23) | 0.811 |
| Nest material | *Staphylococcus*/*Streptococcus* | 0.012 | 0.841 (1,23) | 0.369 |
| Nest material | *E. coli/S. saprophyticus* | 0.005 | 0.325 (1,23) | 0.574 |
| Probability of obtaining 0 of 6 significant tests with p < 0.05 by chance alone: p = 0.735 | | | | |

^a^ Values in parentheses represent degrees of freedom of the numerator and denominator, respectively.

1. Moran MD (2003) Arguments for rejecting the sequential Bonferroni in ecological studies. Oikos 100: 403-405.
